# Discovery of compounds with viscosity-reducing effects on biopharmaceutical formulations with monoclonal antibodies

**DOI:** 10.1016/j.csbj.2022.09.035

**Published:** 2022-09-26

**Authors:** Matic Proj, Mitja Zidar, Blaž Lebar, Nika Strašek, Goran Miličić, Aleš Žula, Stanislav Gobec

**Affiliations:** a University of Ljubljana, Faculty of Pharmacy, Chair of Pharmaceutical Chemistry, Ljubljana, Slovenia; b Biologics Drug Product, Technical Research and Development, Global Drug Development, Novartis, Lek d.d., Slovenia

**Keywords:** GRAS, Generally Recognized as Safe, HIC, hydrophobic interaction chromatography, mAbs, monoclonal antibodies, MW, molecular weight, PSA, polar surface area, SASA, solvent accessible surface area, SlogP, partition coefficient, VRAs, viscosity-reducing agents, Viscosity, Protein formulations, Biopharmaceuticals, Viscosity-reducing agents, Computational screening

## Abstract

For the development of concentrated monoclonal antibody formulations for subcutaneous administration, the main challenge is the high viscosity of the solutions. To compensate for this, viscosity reducing agents are commonly used as excipients. Here, we applied two computational chemistry approaches to discover new viscosity-reducing agents: fingerprint similarity searching, and physicochemical property filtering. In total, 94 compounds were selected and experimentally evaluated on two model monoclonal antibodies, which led to the discovery of 44 new viscosity-reducing agents. Analysis of the results showed that using a simple filter that selects only compounds with three or more charge groups is a good ‘rule of thumb’ for selecting potential viscosity-reducing agents for two model monoclonal antibody formulations.

## Highlights

• Computational screening yielded 44 new viscosity-reducing agents on two model mAbs.
• Dual excipients for viscosity reduction and solution buffering were discovered.
• Compounds with three or more charges reduce the viscosity of model mAb formulations.
• Filtering based on physicochemical properties can be applied to other mAb formulations.

## Graphical abstract

**Figure:**
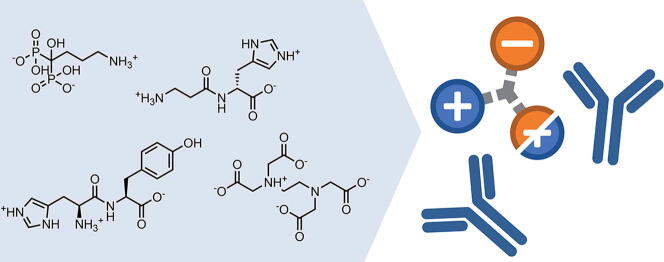

## 1 Introduction

The viscosity of protein solutions increases exponentially with the protein concentration due to numerous noncovalent intermolecular interactions, non-native aggregation, and concentration-dependent fluctuations of various antibody regions [1]. Large transient networks of reversibly associated protein molecules resist flow, and therefore show higher solution viscosity [2]. This presents a major challenge in the formulation development of therapeutic proteins, such as monoclonal antibodies (mAbs), despite their rapid development for pharmaceutical use. High-concentration protein formulations (i.e., low volume) for subcutaneous administration are a sought-after alternative to low-concentration protein formulations (i.e., high volume) administered intravenously. Their development can allow home administration by patients using autoinjectors [3], to relieve the burden on healthcare providers, which is particularly important in the current COVID-19 pandemic.

A common approach for the preparation of concentrated but low-viscosity aqueous protein formulations is the addition of viscosity-reducing agents (VRAs) that can attenuate the attractive protein–protein interactions by binding to specific protein–protein interaction sites. However, the pool of well-established VRAs for subcutaneous administration that are approved by pharmacopoeias and regulatory agencies is relatively small. Salts or organic compounds that contain both hydrophilic and lipophilic moieties are most commonly used. These control the intramolecular interactions between proteins by shielding charges or by interacting with hydrophobic residues on the protein surface [4], [5]. Examples of such compounds are NaCl and some amino acids, such as arginine, lysine, histidine, and proline.

The best performing VRAs, which include arginine in particular, can also have negative effects on the conformational stability of proteins [6]. This can lead to increased protein aggregation and can affect the safety of the product, which is of utmost importance for biopharmaceutical applications. Consequently, some therapeutic proteins in highly concentrated forms remain out of reach for home use.

As the identification of new VRAs is still largely an empirical process that is performed by random screening of compound libraries [7], [8], [9], [10], [11], better understanding of the relationships between structure and viscosity-reducing properties is of great importance. On the other hand, there are numerous databases of compounds and methods for predicting these properties [12]. A tool tailored to biopharmaceutical development using this vast amount of data as an alternative to the empirical trial-and-error approach would accelerate the discovery of new VRAs.

Here, we present the development of a computational filter that can be used to predict alternative VRAs from a list of available excipients that reduce the viscosity of protein solutions. We identified 44 new compounds to have viscosity-reducing effects on biopharmaceutical protein formulations with mAbs.

## 2 Materials and methods

### 2.1 Analysis of model mAbs

For the purpose of in-silico analysis, the 3D structures of both proteins (MW ∼ 150 kDa) were generated by homology modeling. Antibody sequences were used as an input for homology modelling in Maestro program within Schrödinger suite (Release 2021–2, Schrödinger, LLC, New York, NY). Templates for Fv region were selected based on sequence identity. The framework for complete antibody modelling was selected based on IgG type. Hydrophobic interaction chromatography (HIC) retention factors were obtained from the literature [13]. Surface aggregation propensity score was calculated using an in-house implementation of the algorithm using Python and PyMOL [14]. The isoelectric points were obtained from the literature [15]. The net charge was calculated for the protein homology models using PROPKA3. Spatial charge map was calculated for the protein homology models using an in-house implementation of the algorithm using Python and PyMOL [16].

### 2.2 Virtual compound library preparation

#### 2.2.1 Selected experimentally confirmed viscosity reducers

Known VRAs were manually selected from scientific publications and patent applications (Supplementary Excel file, L001–L121) [4], [8], [10], [17], [18], [19], [20], [21], [22], [23], [24], [25]. This provided 121 VRAs that were classified into 33 clusters using the k-means clustering method based on the RDKit molecular fingerprints [26] in the KNIME analytics platform [27]. Up to two VRAs per cluster were manually selected, which resulted in a set of 36 diverse VRAs (Supplementary Excel file, L001–L121). Each selected VRA was used individually as a query for the fingerprint similarity search.

#### 2.2.2 Compounds available from commercial vendors

The largest library was used for virtual screening based on the fingerprint similarity. The existing libraries from commercial vendors Ambinter, Key Organics, eMolecules, Enamine, Life Chemicals, Maybridge, Otava, Princeton BioMolecular Research, Specs, Vitas-M, and UkrOrgSyntez were downloaded in SDF format and merged, and duplicates were removed. A total of 30 million compounds were used without filtering prior to virtual screening.

#### 2.2.3 Compounds available from vendor Chemspace

A Chemspace catalog of compounds in stock (5.4 million compounds) was used for filtering based on physicochemical properties. Compounds known to cause interference in assay systems [28] and compounds with reactive functional groups [29] were removed from the library.

#### 2.2.4 Compounds classified as safe, and compounds already used in clinical trials

Compounds classified as safe were taken from the following public databases: Substances Added to Food (2,571 compounds) [30]; Food Substances Generally Recognized as Safe (GRAS) from the Select Committee on GRAS Substances database (220 compounds) [31]; US Food and Drug Administration inactive ingredients (428 compounds) [32]; and US Environmental Protection Agency Safer Chemical Ingredients List (600 compounds) [33]. Approved drugs were taken from the DrugCentral database (4,044 compounds) [34]. Compounds studied in clinical trials were obtained from a ZINC *in-trial* subset (6,108 *in stock* compounds) [35]. Substances that had been tested in humans but were not approved or studied in trials (e.g., nutraceuticals, many metabolites) were taken from a ZINC *in-man-only* subset (13,306 *in stock* compounds) [35]. All of the compounds were pooled and duplicates were removed, followed by filtering according to physicochemical properties.

#### 2.2.5 Dipeptide library

A library of dipeptides (400 compounds) was assembled from all possible combinations of 20 natural and common amino acids [36].

### 2.3 Fingerprint similarity search

The merged compound library from various commercial vendors was converted into one-dimensional (1D) structural representations using OpenBabel software, as described previously [37]. Briefly, linear fragments with 1–7 atoms were identified in each compound, while ignoring single atom fragments C, N, and O. If the atoms formed a ring, a fragment was terminated. For each of the fragments, the information about the atoms, bonds, and formation of rings was stored in a set so that there was only one of each fragment type. Chemically identical fragments were then identified and only a single canonical fragment was retained. The remaining fragments were assigned a hash number from 0 to 1020, which was used to set a bit in a 1024-bit vector, called a path-based FP2 fingerprint. Finally, a fast search index was created from the fingerprints.

The database was searched for similar compounds to each of the 36 manually selected queries from the known VRAs [37]. The similarity of compounds from the database to each of the queries was expressed using a Tanimoto similarity index: *T = c/(a + b-c)*, where *a* and *b* are the number of bits present in compounds A and B, respectively, and *c* is the number of shared bits by A and B. From the hits with Tanimoto similarity index > 0.7, a sample of 18 diverse compounds was randomly selected for experimental evaluation (Supplementary Excel file, T039–T056).

### 2.4 Filtering based on physicochemical properties

The KNIME analytics platform was used for filtering based on physicochemical properties [27]. Only compounds with molecular weight between 100 Da and 300 Da and SlogP between –2 and 2 (as calculated for neutral species using the RDKit Descriptor Calculation node [26]) were kept from the Chemspace library. Then, the pKa values were calculated using the pKa node, as implemented in the ChemAxon/Infocom JChem KNIME Extensions [38]. A negative charge group was defined for acids with pKa < 6.4, and a positive charge group for bases with pKa > 8.4. Of the 3,351 compounds with one positive and one negative charge group, those with two positive and one negative charge group (36 compounds) or one positive and two negative charge groups (37 compounds) were visually examined. A set of 17 diverse compounds was selected for purchase (Supplementary Excel file, T057–T073).

For the third virtual screening campaign, a library of compounds that are considered safe or that are already in clinical trials was used. For the compounds with SlogP < 2 as calculated for the neutral species using the RDKit Descriptor Calculation node [26], the Total Charge and Total Absolute Charge at pH 6.0 were calculated using the Epik node from the Schrödinger Extensions for KNIME [39]. In addition, the toxicity of the compounds was predicted using the Derek Nexus rule-based expert system [40], and those that showed a “probable” or “certain probability of toxicity” for any of the endpoints were excluded from further evaluation. Compounds with two positive and one negative charge groups or one positive and two negative charge groups were visually inspected, and 10 were selected for purchase (Supplementary Excel file, T074–T083).

Finally, the Epik node [39] was used to calculate sequential pKa values for the dipeptide library. Our goal for this library was to find compounds with properties such that they could act as both VRAs and buffering agents. To ensure sufficient buffering capacity at the target pH of 6.0, compounds with pKa values between 5 and 7 were investigated. Here, 11 compounds with two positive and one negative charge groups or one positive and two negative charge groups were purchased (Supplementary Excel file, T084–T094).

### 2.5 Analysis of the results

For the analysis of the results, several descriptors were calculated with KNIME using the following nodes: RDKit Descriptor Calculation (partition coefficient [SlogP], hydrogen bond acceptors, hydrogen bond donors, rotatable bonds, heavy atoms, rings, fraction Csp^3^); Canvas Molecular Descriptors (molecular weight [MW], polar surface area); Epik (number of charges, net charge, positive charges, negative charges); and QikProp (solvent accessible surface area, volume, predicted Caco-2 cell permeability in nm/s, predicted aqueous solubility) [26], [39]. The correlations between the experimental results and calculated descriptors were analyzed using Pandas, Matplotlib, Seaborn, and Statannot libraries in Python v3.7.10.

### 2.6 Likelihood of toxicity prediction

The knowledge-based expert system Derek Nexus (Lhasa Limited, Leeds, UK) was used to estimate the toxicity of the compounds [40]. This software can provide useful structural alert information to help in compound selection. When batch prediction mode was set up, all of the endpoints and the following species were selected for the report: bacterium, *Salmonella typhimurium*, mammal, and human. The likelihood of prediction was set to at least plausible; i.e., plausible, probable, and certain. If no alerts were triggered with a probability of plausible or higher, the test compound was classified as nontoxic.

### 2.7 Sample preparation

Selected mAb dispersions were provided by Lek Pharmaceuticals d.d., The protein material was dialyzed against purified water (Purelab Chorus). The pHs of the protein stock solutions in water were adjusted to the target pH value of pH 6.0 using low-concentration HCl or NaOH (with no adverse effects on the proteins, as confirmed by size-exclusion chromatography). Protein stocks were then concentrated in tubes (ultra-centrifuge filter units, 50 kDa MW cut-off; Amicon) to a protein concentration of approximately 180 mg/mL. The concentrations were measured in triplicate after dilution with water to approximately 1 mg/mL. In parallel, stock solutions of VRAs were prepared in purified water and their pHs were adjusted to the target pH of 6.0. The VRAs in the stock solutions were prepared at concentrations 1.5-fold higher than the final 25 mM concentration, because these were diluted in the next step. At this stage, the protein stock and VRA stock solutions were mixed volumetrically in a 1:2 ratio to obtain samples with a protein concentration of 60 mg/mL. To obtain the final samples with a protein formulation of 150 mg/mL, a concentration step of all of the samples using the ultra-centrifuge filter units was required (ultra-centrifuge filter units, 10 kDa MW cut-off; Amicon). The pH increased by 0.2 during up-concentration from 60 mg/mL to 150 mg/mL. The differences in concentrations in retentate and permeate were < 10 % for some representative excipients (i.e., sucrose, histidine, and NaCl). As those excipients represent a range of interactions, similar was assumed for all other excipients. This approach to prepare the final samples with 25 mM VRAs and a protein concentration of ∼ 150 mg/mL was the most feasible, because in some cases the required VRA concentration exceeded the water solubility for direct mixing. The prepared samples had similar pHs, and the errors in the concentration measurements (estimated from triplicate measurements at 1 %) were also considered.

### 2.8 Viscosity measurement

Viscosity was measured with a viscometer–rheometer (RheoSense VROC [viscometer-rheometer-on-a-chip]) that uses microfluidic technology. The chip used had a 2 mm × 50 mm × 13 mm rectangular slit microfluidic channel. All of the viscosity measurements were performed at 25 °C. The shear rate was varied between 2,000 s^−1^ and 6,000 s^−1^ to retain constant pressure in the channel. The protein solutions were previously measured as Newtonian in this range (i.e., viscosity changed by 0.2 cP or about 1 % in this range). The protein concentration determines the viscosity of the protein in water without excipients. Therefore, the baseline viscosity of protein in water was measured in the appropriate concentration range (120–200 mg/mL). An exponential function $\eta =a*e^{b*c}$, where η is the viscosity of protein in water and c is the protein concentration, was fitted to the data (Fig. S3). Before each viscosity measurement of protein with added VRA (η), the exact protein concentration was determined spectrophotometrically at 280 nm. The baseline viscosity of protein in water ($\eta_{0}$) was calculated for this concentration using the exponential fit. Finally, relative viscosity ($\eta /\eta_{0}$) was obtained.

## 3 Results

Two model IgGs were selected to be generally representative of all therapeutic mAbs. The main considered criteria were hydrophobicity, charge, and charge distribution. HIC retention factors of mAbs A and B are around 4 and 2, respectively. In comparison to other therapeutic mAbs, mAb A can be considered hydrophobic and mAb B hydrophilic [13]. This is confirmed by calculation of the surface aggregation propensity score [14] of the protein homology models. The surface aggregation propensity score projected to both protein surfaces (Fig. S1A, B) revealed that mAb A has several hydrophobic patches in and around the complementarity determining region. In contrast, mAb B has no significant hydrophobic patches on the Fab. Overall, the total surface aggregation propensity score of mAb A is higher by 5 points, which means a significantly larger contribution of hydrophobic interactions to self-association. The isoelectric point of mAb A is above pH 9, and the isoelectric point of mAb B is below pH 8 (relative difference between them is 1.5 units), which again puts both mAbs on the opposite sides of the distribution of mAb properties [15]. At pH 6.0, mAb A has nearly twice the net charge of mAb B as calculated with PROPKA3 (Fig. S2). Next, we inspected charge distribution on the protein surface (Fig. S1C, D). mAb A is mostly positively charged, while both negatively as well as positively charge surface patches can be seen on the mAb B. The latter has especially prominent negative patches near the complementarity determining region, which have been shown to correlate strongly with increased viscosity [16].

**Fig. 1:**
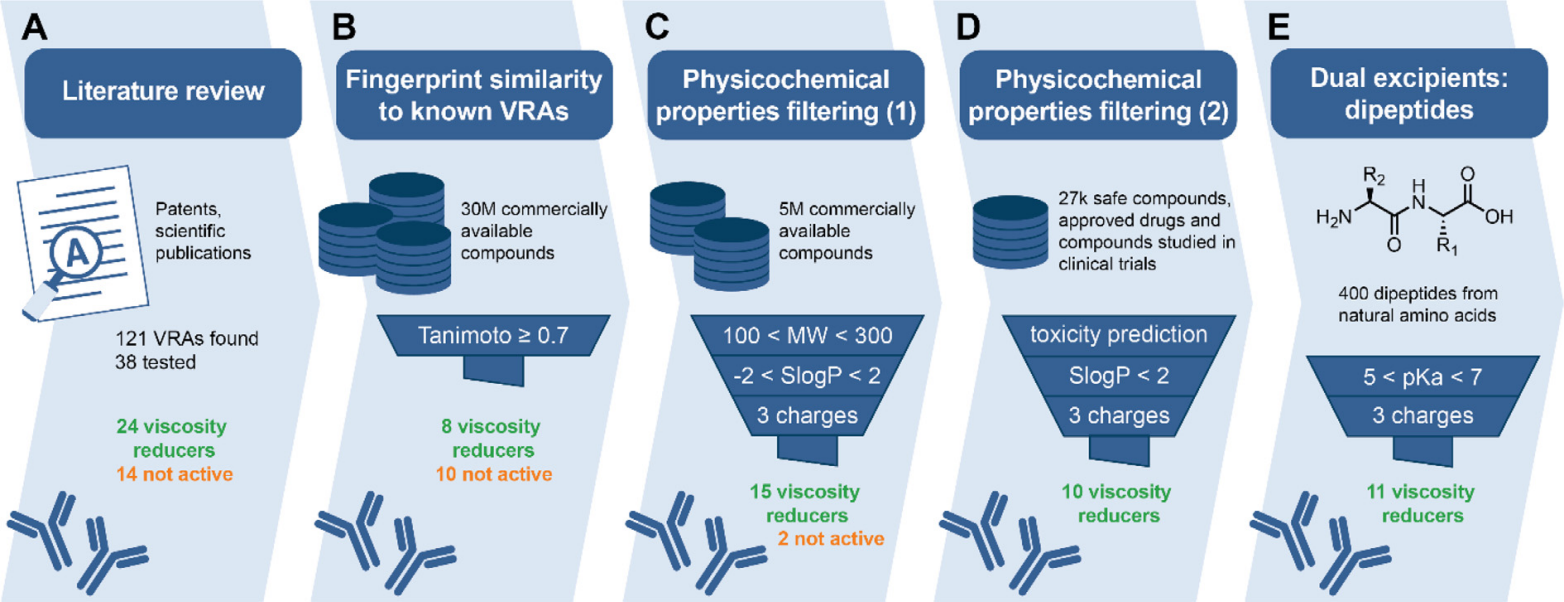
Workflow used to identify new viscosity-reducing agents. A different source of compounds and a different filter were used for each step. The viscosity of two model mAb solutions was measured in the presence of each compound tested. Overall, 68 of 94 compounds had a viscosity-reducing effects.

The targeted protein concentration for preparation of formulation was 150 mg/mL and the viscosities of mAb A and mAb B were 10–30 cP at this concentration (Fig. S3). According to a study by Berteau et al., solutions for subcutaneous administration with viscosity up to 20 cP are well tolerated without pain. Administration of solutions with higher viscosity not only causes pain but also requires forces that are too high for self-administration with a needle size commonly used for subcutaneous administration (i.e., thinner than 27 gauge) [41]. Therefore, our target viscosity range was below 20 cP, since we considered this as the syringeability limit. In this viscosity range, arginine as currently the best VRA in general use reduces the viscosity by 40–50 % (relative viscosity 0.5–0.6) at 25 mM. Considering the experimental error arising from both viscosity and protein concentration measurements (around 0.1 for relative viscosity), we set the threshold for classification of a compound as a VRA at an average relative viscosity for two model mAbs of ≤ 0.8. This is a reasonable limit within our experimental setup to detect VRAs with similar viscosity reducing effects as arginine.

Review of patents and scientific literature revealed 121 known VRAs (Supplementary Excel file, L001–L121). The available compounds from our in-house chemical library were tested on two model mAbs. Less than two-thirds of the 38 compounds from the literature that were tested (Supplementary Excel file, T001–T038) had viscosity-reducing effect in our test systems (Fig. 1A). This demonstrates that not all VRAs are universally applicable and highlights the importance of expanding the chemical space of known VRAs to facilitate the development of new mAb formulations.

Ligand-based drug design is a commonly used approach in drug discovery that uses a known compound as a query to find similar compounds. This is based on the concept that structurally similar compounds tend to have similar biological properties [42]. Accordingly, our first virtual screening campaign was a search for compounds similar to known VRAs. First, 36 diverse query compounds were selected from the known VRAs. Second, we performed a fingerprint similarity search in which the 2D chemical structure was fragmented and converted into a bit sequence. This linear (1D) representation of each query compound was compared with each compound from the library of 30 million commercially available compounds. Hits with a Tanimoto similarity index of ≥ 0.7 to any of the queries were considered as similar [37]. A sample of 18 diverse compounds (Supplementary Excel file, T039–T056) was then randomly selected for experimental evaluation. Almost half of the compounds tested reduced the solution viscosity of two model mAbs (Fig. 1B).

To rationalize these results, we set filters for the next step of computational screening. Filtering based on molecular descriptors such as the Lipinski rule-of-five is commonly used in drug design to increase the likelihood of selecting water-soluble and permeable compounds [43]. We hypothesized that a similar approach could be applied to the selection of compounds that have the potential to reduce the viscosity of protein solutions. Therefore, we investigated the relationships between the average relative viscosity of two model mAbs and three basic molecular descriptors.

**Fig. 2:**
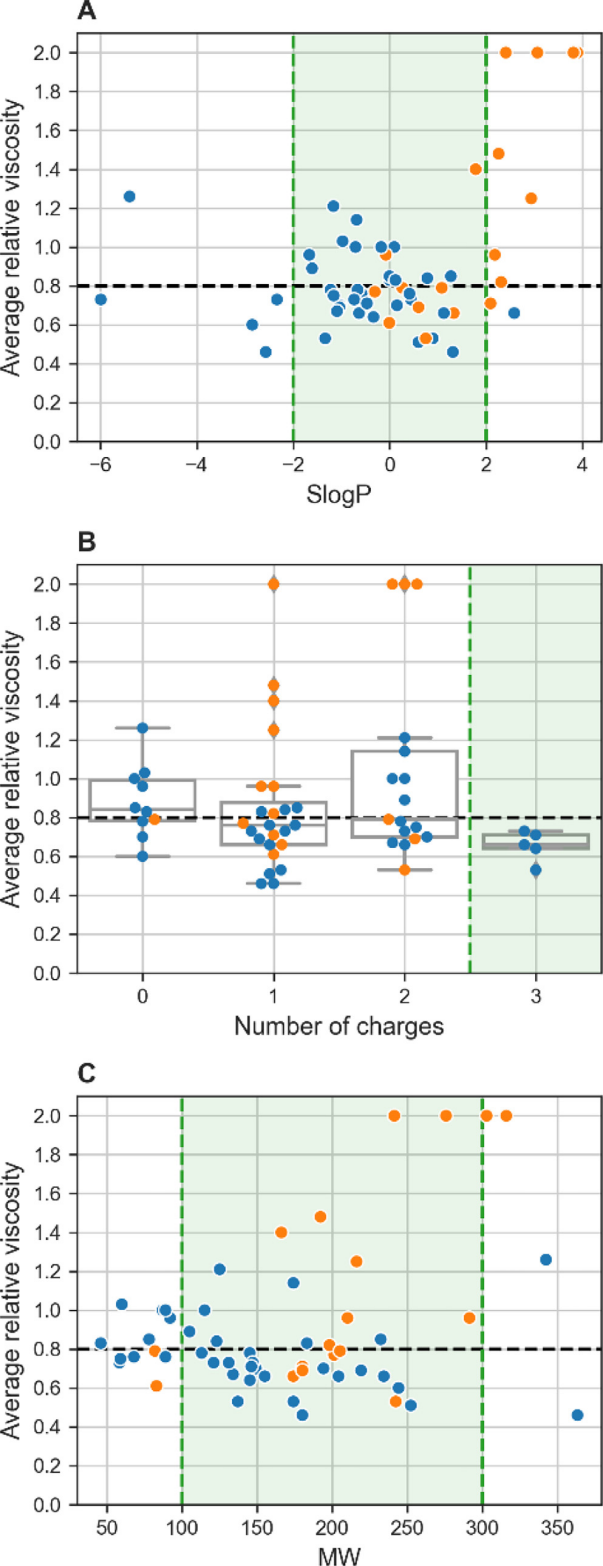
Analysis of experimental results for known viscosity-reducing agents (blue) and compounds obtained by fingerprint similarity screening (orange) (N = 56). Relationships between average relative viscosity and three basic molecular descriptors are shown, for SlogP (**A**), number of charge groups at pH 6.0 (**B**), and molecular weight (MW) (**C**). Black dashed lines represent threshold for viscosity reduction; green areas represent selected values used for filtering in the next step. (For interpretation of the references to colour in this figure legend, the reader is referred to the web version of this article.)

The first descriptor was the partition coefficient logP, as this is important for drug design. We used a method implemented in RDKit to estimate the logP value (i.e., SlogP) by summing the contributions of atom-weighted solvent-accessible surface areas and correction factors [44]. As most of the viscosity-reducing compounds were in the range between –2 and 2 SlogP (Fig. 2A), we used this range as a filter for the next step. Second, it was known from previous studies that the masking of charges on the protein surface is an important mechanism for preventing protein–protein interactions and reducing the viscosity of a solution [45]. When we examined the correlation of viscosity with the number of charge groups, all compounds with three charge groups reduced the viscosity of protein solutions (Fig. 2B). Therefore, compounds with less than three charge groups would be sorted out in the next step. Third, although MW is only lightly correlated with viscosity reduction (Fig. 2C), we arbitrarily limited the MW to 100 Da to 300 Da, to retain only simple compounds. In addition, the combination of filters chosen should ensure good water solubility of the compounds.

**Fig. 3:**
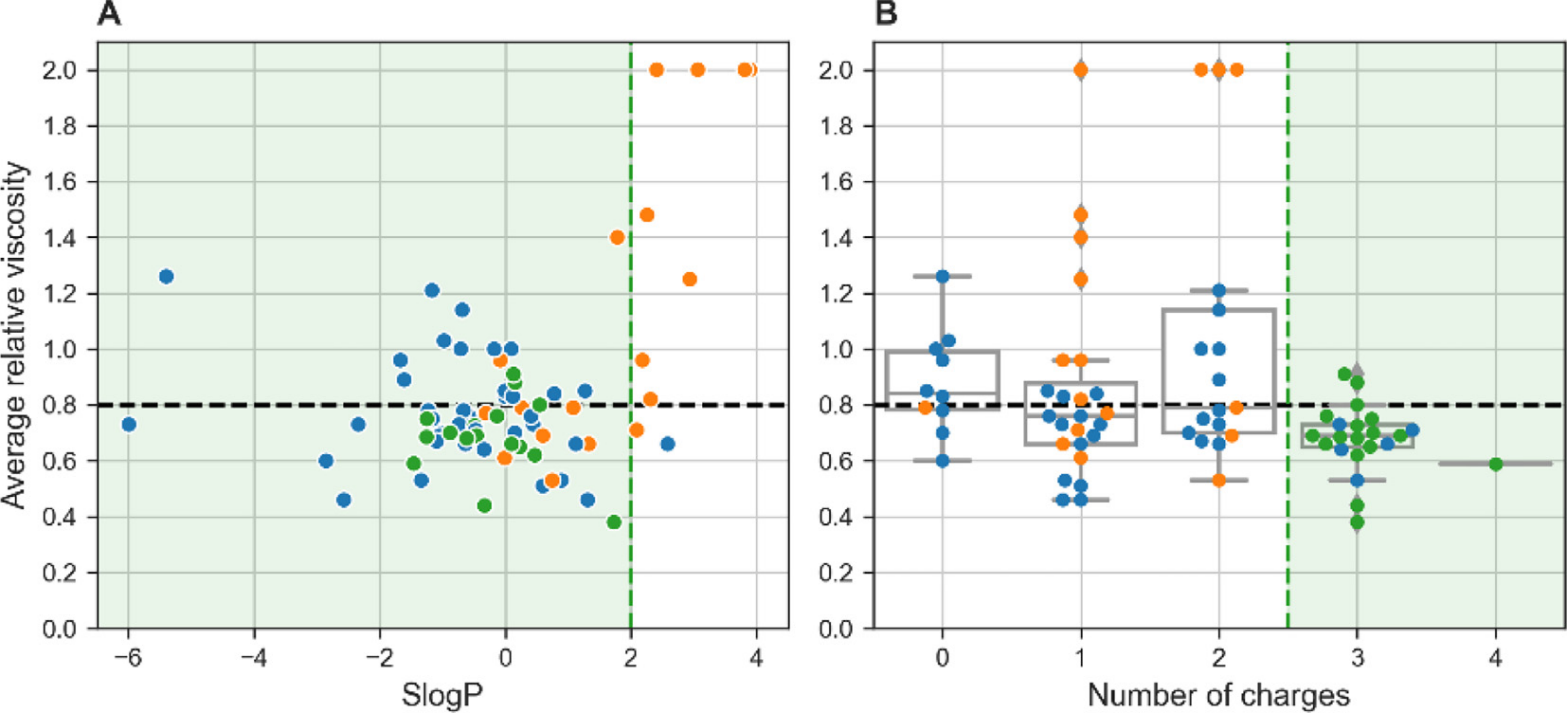
Average relative viscosity results for known viscosity-reducing agents (blue), similar compounds based on fingerprint similarity (orange), and filtered compounds from the Chemspace library (green) (N = 73). The relationships between average relative viscosity and SlogP (**A**) and average relative viscosity and number of charge groups at pH 6.0 (**B**) are shown. Black dashed lines represent threshold for viscosity reduction; green areas represent selected values used for filtering in the next step. (For interpretation of the references to colour in this figure legend, the reader is referred to the web version of this article.)

To identify new viscosity reducers with novel scaffolds that are structurally less similar to the already known VRAs but still share the same key physicochemical properties, we applied the filters selected above to the Chemspace library of in-stock compounds (5.4 million). Therefore, only compounds with SlogP between –2 and 2, at least three charge groups at pH 7.4, and MW between 100 Da and 300 Da were considered. This resulted in 73 compounds that met the criteria, which were then visually inspected. We selected 17 diverse compounds for experimental evaluation (Supplementary Excel file, T057–T073), and 15 of these were shown to reduce solution viscosity (Fig. 1C). Encouraged by these results, we wanted to determine which was the most important descriptor and to establish rules of thumb that could be used for rapid filtering of libraries containing approved or safe excipients. Therefore, we set less stringent filters with cut-off values as shown in Fig. 3 to be used in the next step. In particular, the lower cut-off value for SlogP of > –2 was omitted, as was the filter for MW, which, as mentioned earlier, is only minimally correlated with the reduction in viscosity. The most important observation here was that the majority of compounds with three or more charge groups reduced mAb solution viscosity (Fig. 3B).

Our next aim was to reduce risk of unwanted toxicity for novel excipients when going from *in* vitro to *in* vivo studies. Therefore, we compiled a library of safe compounds [30], [31], [32], [33], compounds studied in clinical trials [35], and approved drugs [34] for the third virtual screening campaign. Of the original 27,277 compounds, we selected only those with SlogP < 2 and three or more charge groups at pH 6.0, taking advantage of our experiences from the previous two campaigns. In addition, the toxicities of selected compounds were predicted using the Derek Nexus [40] rule-based expert system and those that were likely to be toxic were excluded from further evaluation. Ten compounds were tested (Supplementary Excel file, T074–T083), and viscosity-reducing capabilities were experimentally confirmed for all of them (Fig. 1D).

Finally, we have turned to another source of safe and naturally occurring compounds. There are only five natural amino acids with three charge groups at physiological pH: arginine, histidine, lysine, glutamic acid, and aspartic acid. We confirmed the viscosity-reducing effects for all of them (Supplementary Excel file, T003, T010, T015, T016, T080). Moreover, histidine is commonly used as a buffer in highly concentrated biopharmaceutical formulations [46]. To expand the chemical space of amino acids, we assembled a library of 400 dipeptides from all possible combinations of 20 natural and common amino acids. We selected dipeptides with at least one pKa value between 5 and 7, which we assumed would have a high buffering capacity at the targeted pH of 6.0. Of these dipeptides, 11 dipeptides with three or more charge groups were tested (Supplementary Excel file, T084–T094). Again, all these compounds were found to reduce the viscosity of protein solutions (Fig. 1E). This result further confirms the importance of three charge groups in reducing the viscosity of our mAb formulations.

## 4 Discussion

In most VRA studies in the literature, a buffer is added to the sample in addition to the compound of interest. However, various buffer components in combination with the VRAs tested are expected to have nonadditive effects on solution viscosity [47]. In our study, we relied on the self-buffering effect of the highly concentrated protein solution [48], which eliminated the need for an additional buffer in the formulation. Therefore, viscosity-reducing effects of compounds can be unambiguously deduced from the results.

**Fig. 4:**
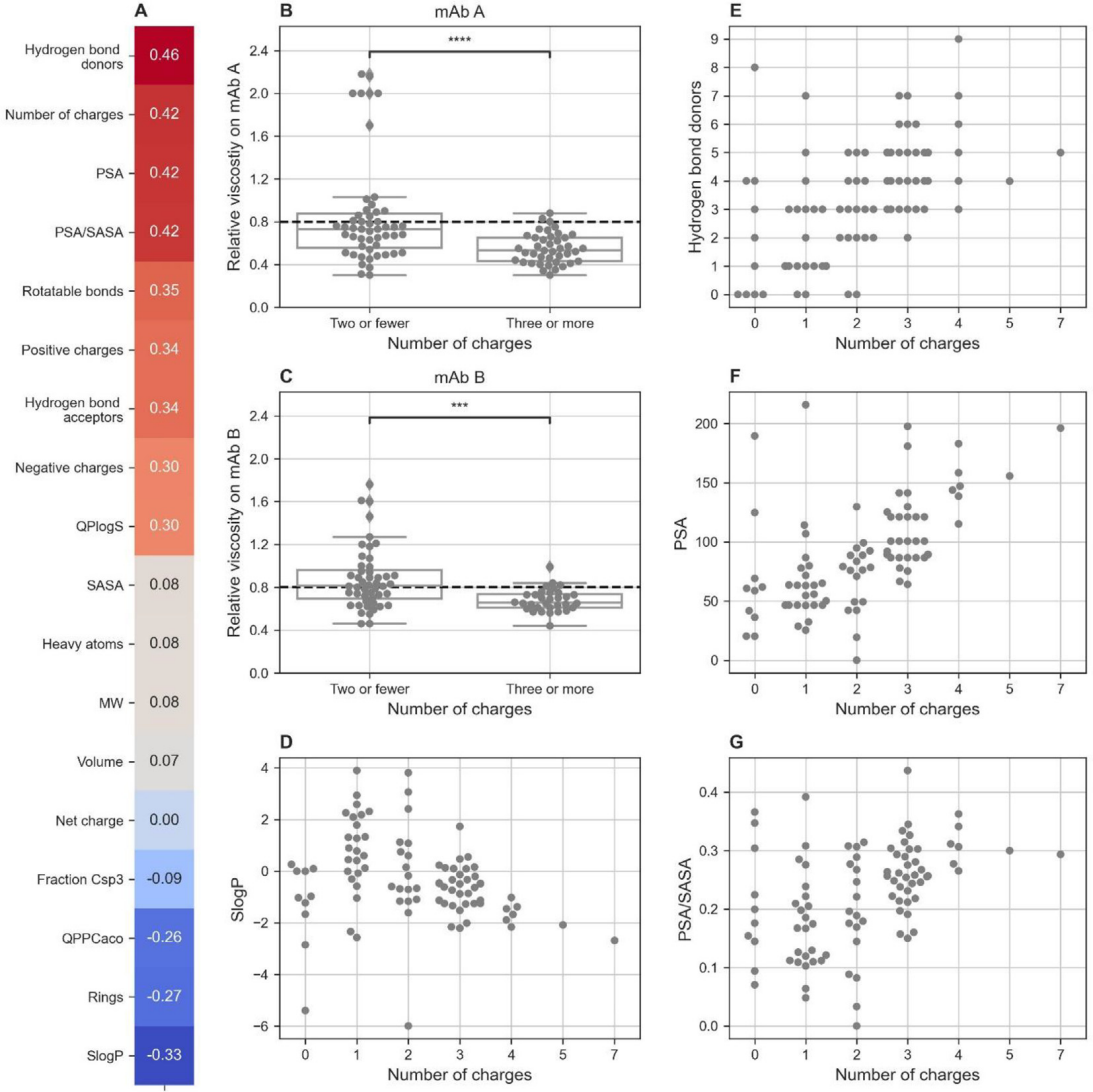
Analysis of all of the experimental results (N = 94). (**A**) Pearson correlation coefficients between the viscosity reducer classifier with a threshold average relative viscosity of 0.8 and several descriptors. (**B**, **C**) Compounds with three or more charge groups significantly reduced the relative viscosity of two model mAb formulations, compared to compounds with two or fewer charge groups. ***, p ≤ 0.001; ****, p ≤ 0.0001 (two-tailed Mann–Whitney–Wilcoxon tests). Black dashed lines, threshold for viscosity reduction. (**D**) The relationship between SlogP and the number of charge groups for our series of compounds. (**E**) The relationship between the number of hydrogen bond donors and the number of charge groups for our series of compounds. (**F**) The relationship between PSA and the number of charge groups for our series of compounds. (**G**) The relationship between PSA/SASA and the number of charge groups for our series of compounds.

Overall, a total of 94 compounds were evaluated experimentally (Supplementary Excel file, T001–T094). For analysis, compounds were categorized as those that decreased or did not decrease the solution viscosity. To detect all VRAs with similar viscosity reducing effects as arginine, the threshold was set to an average relative viscosity for two model mAbs ≤ 0.8. Pearson correlation coefficients were calculated between this binary classifier and several molecular descriptors. Analysis of all 94 compounds revealed that the number of charge groups was indeed strongly correlated with the decrease in viscosity of the protein solution (Fig. 4A). Moreover, compounds with three or more charge groups significantly reduced the relative viscosities of the two model mAb formulations, compared with compounds with two or fewer charge groups (Fig. 4B, C). Filtering based on logP, which was used in the initial screenings, was not necessary because three or more ionizable functional groups already contributed sufficiently to the polarity of the compounds, as can be seen from the series of compounds in Fig. 4D. The descriptors that most strongly correlated with the decrease in viscosity of the protein solution (Fig. 4A), namely hydrogen bond donors, number of charge groups, polar surface area (PSA), and polar surface area relative to solvent accessible surface area (PSA/SASA) are not independent. Ionizable functional groups contain hydrogen bond donors and increase PSA and PSA/SASA as confirmed by the correlation with the number of charged groups for the series of compounds in Fig. 4E, F, G.

From these observations, we can conclude that the number of charge groups is the most important descriptor and can be used as a rule of thumb to filter out compounds that are likely to reduce viscosity for two model mAbs. We should note here that the application of this rule of thumb excludes all viscosity reducers with two or fewer charge groups already described in the literature. Nevertheless, the high values of the metrics derived from the error matrix confirm that this approach has acceptable predictive power (Table S1). For example, filtering by charged compounds is most appropriate when we have a list of potential excipients and want to narrow it down to a few excipients that have a high probability of reducing the viscosity of mAb formulations.

As *in-silico* prediction of toxicity was used as a filter in only one of the screening campaigns, the toxicities of all remaining compounds were also estimated using the Derek Nexus knowledge-based expert system. The software identifies substructures or fragments of compounds that may cause toxicity by applying a database of rules compiled by toxicologists and experts. The rules and relationships are based on empirical observations. The absence of an alert indicates that toxicity is less likely [49]. There were only four compounds with a “probable” or “certain probability of toxicity” (i.e., acetylsalicylic acid, caffeine, ethanol, *N*,*N*-dimethylacetamide), and all four have been previously used as VRAs in the literature [10], [22], [24], [50]. Another nine VRAs from the literature were classified as having a “plausible probability of toxicity”. In contrast, of the newly discovered VRAs from the virtual screenings, 16 out of 44 compounds were classified as having a “plausible probability of toxicity” (see Supplementary Excel file for structural alerts and toxicological endpoints). This indicates that the majority of the newly discovered VRAs appear to have unproblematic toxicological profiles.

**Fig. 5:**
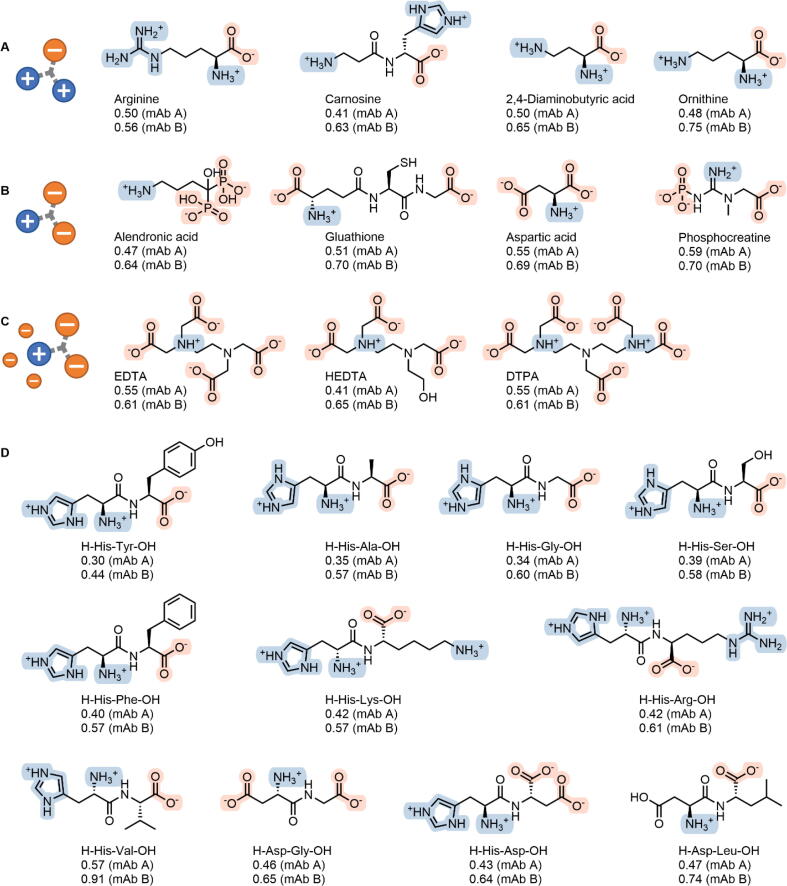
Examples of newly discovered viscosity reducers from databases of safe compounds, compounds studied in clinical trials, and approved drugs with relative viscosity for two model monoclonal antibodies, compared with arginine. The threshold for classification of a compound as a viscosity reducer was set at an average relative viscosity ≤ 0.8 for two model monoclonal antibodies. The charges of the major species at pH 6.0 are shown. (**A-C**) Based on the number of charge groups and considering the predominantly positive charge on the protein surface at 2–3 units below the isoelectric point, we propose three different mechanisms for viscosity reduction: neutralization of negatively charged regions (**A**); neutralization of predominantly positive charges (**B**); and charge reversal (**C**). EDTA, ethylenediaminetetraacetic acid; HEDTA, hydroxyethylethylenediaminetriacetic acid; DTPA, diethylenetriaminepentaacetic acid. (**D**) Structures of dipeptides that can be used as dual excipients for viscosity reduction and solution buffering.

However, to avoid overestimating the predictive power of the *in-silico* estimated toxicity, we specifically wanted to highlight only VRAs with known safety profiles. Therefore, ten newly discovered VRAs were selected: four from databases of safe compounds (aspartic acid, ethylenediaminetetraacetic acid, hydroxyethylethylenediaminetriacetic acid, and diethylenetriaminepentaacetic acid), three studied in clinical trials (carnosine, 2,4-diaminobutyric acid, and ornithine), and three approved drugs (alendronic acid, glutathione, and phosphocreatine) (Fig. 5A-C).

In addition, 11 dipeptides in which one of the side chains was basic or acidic were shown to decrease the viscosity of the mAb solution (Fig. 5D). Given their pKa values, the dipeptides tested were expected to have a high buffering capacity at pH 6.0, similar to histidine, and could thus be used as ‘dual excipients’ for viscosity reduction and solution buffering. This would reduce the number of excipients needed and simplify the development of new mAb formulations. Overall, the compounds shown in Fig. 5 can be readily used as excipients for the development of highly concentrated mAb formulations.

Differences in mAb viscosities are largely due to differences in hydrophobic and electrostatic interactions [47]. Therefore, hydrophobicity, charge, and charge distribution were considered in the selection of model mAbs to be representative of all therapeutic mAbs. Under the specified experimental conditions (i.e., pH 6.0), mAb A has several exposed hydrophobic patches but has a high net charge leading to electrostatic repulsion. In contrast, mAb B is much less hydrophobic but has a low net charge. We conclude that attractive protein–protein interactions in mAb A are mostly hydrophobic in nature, while in mAb B they are caused by electrostatic interactions between oppositely charged patches. This implies that different factors contribute to the increase in viscosity of our model mAbs solutions. At this point, it should be noted that if the pH is lowered further (e.g., by 4 or more units below the isoelectric point), the net electrostatic effects between protein molecules alone should reduce the viscosity [47]. Therefore, the conclusions presented here cannot be generalized to the entire pH range, but only to a range of pH values around the isoelectric point for globular proteins.

## 5 Conclusions

Here, we present the use of computational filters to single out compounds that can reduce the viscosity of monoclonal antibody solutions. Two computational approaches were used: searching for fingerprint similarities, and filtering based on physicochemical properties. In this way, 33 new compounds with viscosity-reducing effects on two model mAbs were identified. In addition, 11 dipeptides were discovered that can be used as dual excipients to simultaneously reduce viscosity and buffer the solution. Most importantly, we discovered that compounds with three or more charge groups at pH 6.0 have a high potential to reduce the viscosity of model mAbs. Our iterative approach of filtering compounds based on physicochemical properties is a simple and efficient strategy that can be applied to other mAb formulations to identify new VRAs.

## CRediT authorship contribution statement

**Matic Proj:** Investigation, Writing – original draft. **Mitja Zidar:** Investigation, Validation. **Blaž Lebar:** Investigation. **Nika Strašek:** Investigation. **Goran Miličić:** Investigation. **Aleš Žula:** Resources, Validation, Writing – review & editing. **Stanislav Gobec:** Writing – review & editing, Supervision.

## Declaration of Competing Interest

The authors declare that they have no known competing financial interests or personal relationships that could have appeared to influence the work reported in this paper.
