# Supplementary material for: Discovery of compounds with viscosity-reducing effects on biopharmaceutical formulations with monoclonal antibodies
Source: Comput Struct Biotechnol J. 2022 Sep 26;20:5420–9. doi: 10.1016/j.csbj.2022.09.035 (PMC9529560; doi:10.1016/j.csbj.2022.09.035)
Supplement: Supplementary data 2 [file mmc2.docx]

**Supplementary information**

**Discovery of compounds with viscosity-reducing effects on biopharmaceutical formulations with monoclonal antibodies**

Matic Proj ^a,+^, Mitja Zidar ^b^, Blaž Lebar ^a^, Nika Strašek ^a^, Goran Miličić ^b^, Aleš Žula ^b,+,^*, Stanislav Gobec ^a,^*

^a^ *University of Ljubljana, Faculty of Pharmacy, Chair of Pharmaceutical Chemistry, Ljubljana, Slovenia*

^b^ *Biologics Drug Product, Technical Research and Development, Global Drug Development, Novartis, Lek d.d., Slovenia*

^+^ *Both authors contributed equally to this work.*

** Corresponding authors. E-mail addresses:* [stanislav.gobec@ffa.uni-lj.si](mailto:stanislav.gobec@ffa.uni-lj.si) (S. Gobec), [ales.zula@novartis.com](mailto:ales.zula@novartis.com) (A. Žula).

**Table of contents**

[1 Supplementary figures and tables S2](#_Toc104460927)

[2 Supplementary references S4](#_Toc104460928)

## Supplementary figures and tables


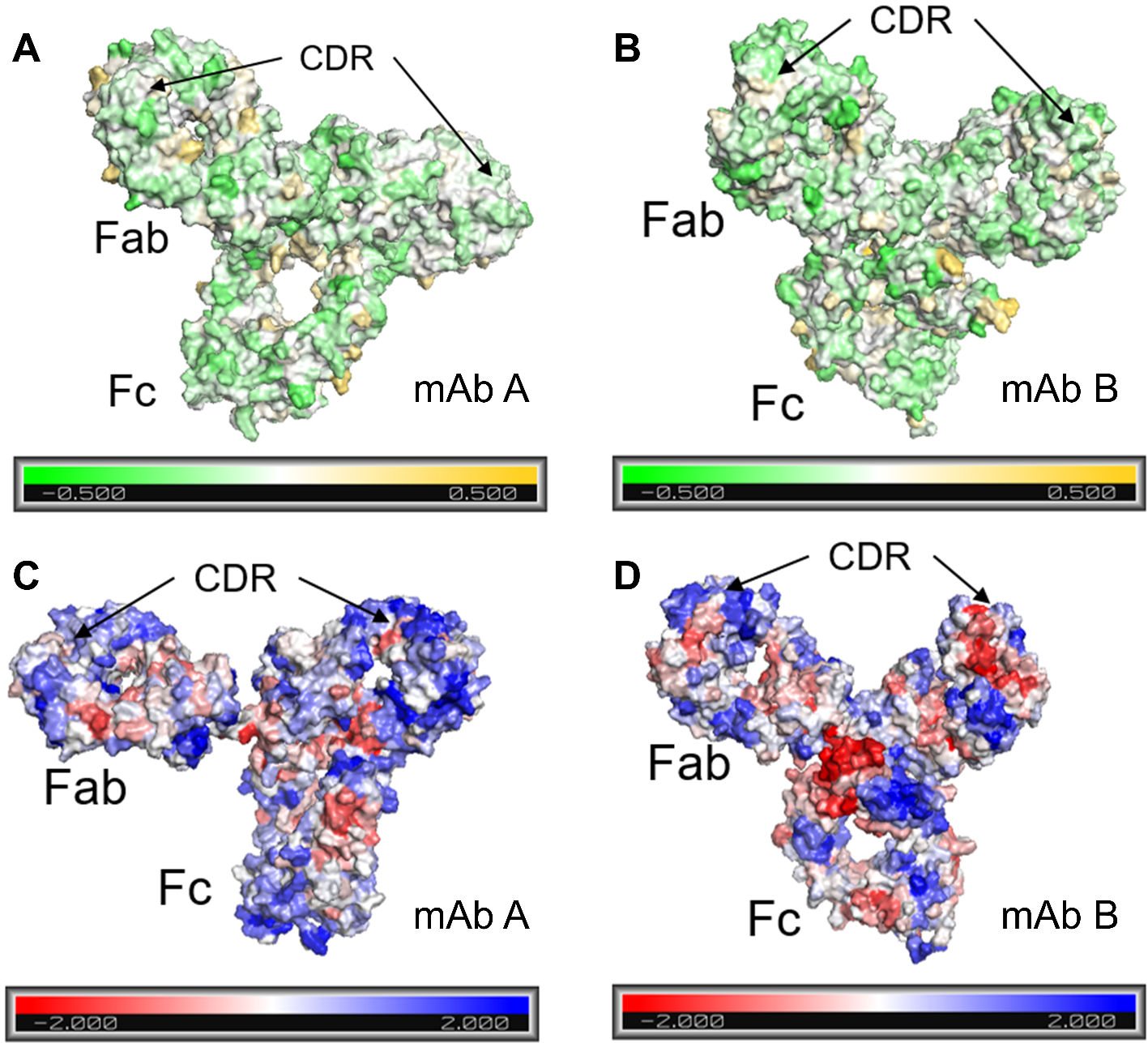


**Fig. S1**. (**A**–**B**) Surface aggregation propensity score [1] projected on both protein surfaces. Yellow color indicates hydrophobic patches, and green indicates hydrophilic patches. (**C**–**D**) Spatial charge map [2] of both proteins. Blue color indicates positively charged patches, and red color indicates negatively charged patches.


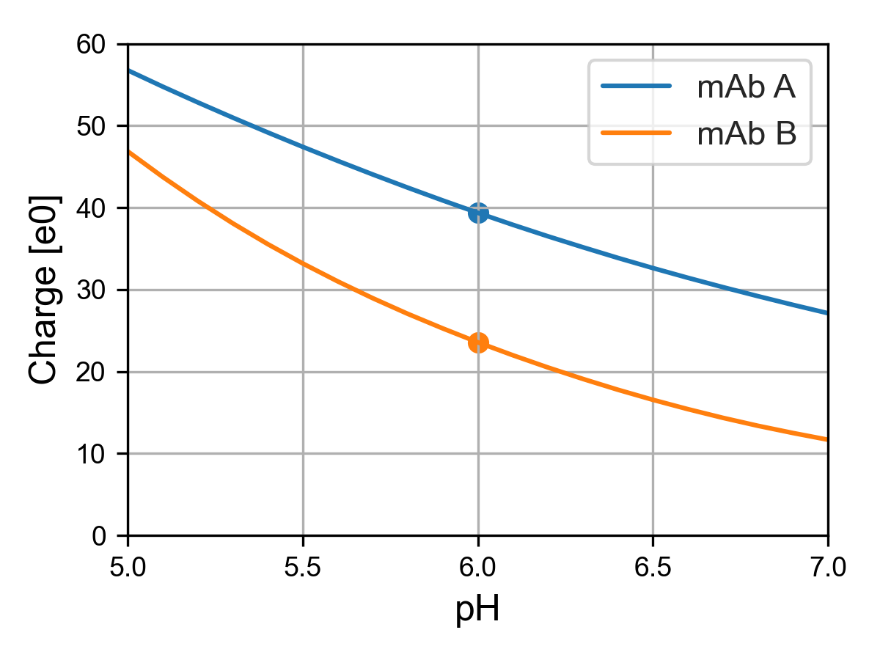


**Fig. S2**. Net charge as a function of pH calculated with PROPKA3 for both proteins. At the targeted pH 6, the net charge of mAb A is 40 e_0_, and the charge of mAb B is 24 e_0_.


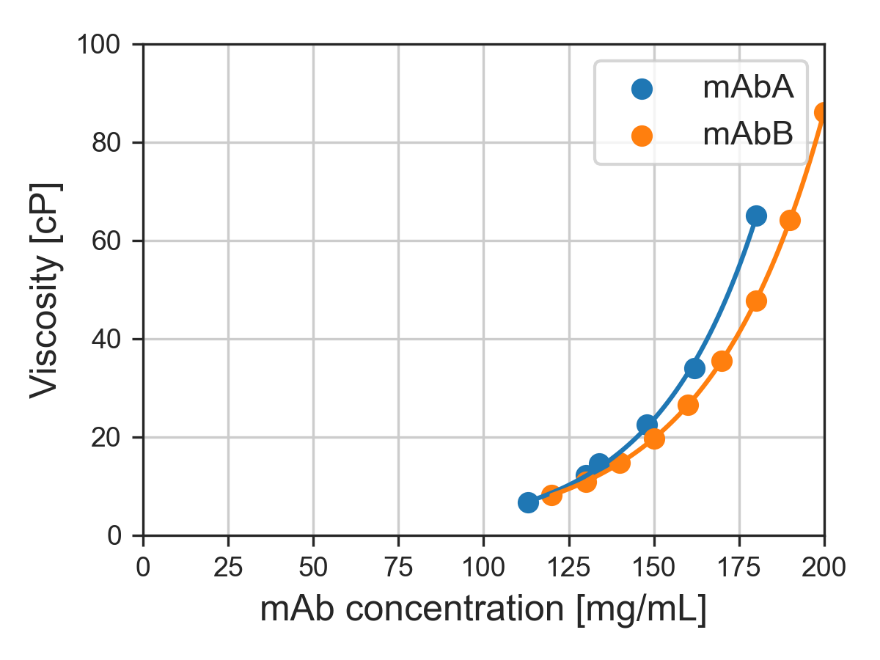


**Fig. S3**. Baseline viscosity for both proteins in water as a function of protein concentration. An exponential function $\eta=a*e^{b*c}$, where $\eta$ is the viscosity of protein in water and $c$ is the protein concentration, was fitted to the data.

**Table S1**. Error matrix (i.e., contingency table) describing the performance of the rule of thumb for all of the compounds tested (N=94). Sensitivity, 0.59; accuracy, 0.68; specificity, 0.92; precision, 0.95; F-score, 0.73.

| **Average relative viscosity <0.8** | **Number of charge groups** | |
| --- | --- | --- |
|  | **Two or fewer** | **Three or more** |
| No effects on viscosity | 24 | 2 |
|  | (true negatives) | (false positives) |
| Viscosity-reducing agent | 28 | 40 |
|  | (false negatives) | (true positives) |

## Supplementary references

[1] N. Chennamsetty, V. Voynov, V. Kayser, B. Helk, B.L. Trout, Design of therapeutic proteins with enhanced stability, Proc. Natl. Acad. Sci. 106 (2009) 11937–11942. https://doi.org/10.1073/pnas.0904191106.

[2] N.J. Agrawal, B. Helk, S. Kumar, N. Mody, H.A. Sathish, H.S. Samra, P.M. Buck, L. Li, B.L. Trout, Computational tool for the early screening of monoclonal antibodies for their viscosities, MAbs. 8 (2016) 43–48. https://doi.org/10.1080/19420862.2015.1099773.
